# Regional gray matter volume correlates to physical and mental fatigue in healthy middle-aged adults

**DOI:** 10.1016/j.ynirp.2022.100128

**Published:** 2022-09-02

**Authors:** Handityo Aulia Putra, Kaechang Park, Fumio Yamashita, Kei Mizuno, Yasuyoshi Watanabe

**Affiliations:** a Research Organization for Regional Alliances, Kochi University of Technology, Kochi, Japan; b Department of Engineering, Keimyung University, Daegu, Republic of Korea; c Division of Ultrahigh Field MRI, Institute for Biomedical Sciences, Iwate Medical University, Iwate, Japan; d Laboratory for Pathophysiological and Health Science, RIKEN Center for Biosystems Dynamics Research, Kobe, Japan

**Keywords:** Physical fatigue, Mental fatigue, Regional gray matter volume, Chalder's fatigue questionnaire, Middle-aged adults

## Abstract

Previous studies on neural/molecular mechanisms of fatigue focused on a variety of brain functions, morphological changes, and neurochemical functions such as neurotransmitter and neuroimmune dynamics. However, MRI morphological changes were adopted primarily to compare patients with Myalgic Encephalomyelitis/Chronic Fatigue Syndrome (ME/CFS) and healthy controls. A few studies have been done on healthy subjects with fatigue scores; one study with 63 adults (their ages of 53.2 ± 8.3) showed the gray matter volume (GMV) reduction in good correlation with a higher score of fatigue. The other one with university students (their ages of 20.7 ± 1.8) demonstrated no significant correlation between regional GMV (rGMV) and fatigue severity. To elucidate the brain structural underpinning in parallel with fatigue development, a large number of healthy middle-aged adults (n = 1873; aged 54.1 ± 5.4) without ME/CFS were recruited, and the correlation between both rGMV in the cerebrum including basal ganglia and Chalder's fatigue questionnaire (CFQ) with physical and mental categories were investigated. A higher CFQ score denotes a higher perceived fatigue level by the participant. The physical fatigue scores of CFQ showed a significantly negative correlation (i.e., smaller rGMV for higher CFQ score) with the volume of the right planum temporale and supplemental motor cortex (SMC), while the left putamen, middle temporal gyrus (MTG), parietal operculum, and right precentral gyrus showed a significantly positive correlation (i.e., bigger rGMV for higher CFQ score). In the mental fatigue scores, the right SMC and left lateral orbital gyrus (LOG) showed a significantly negative correlation, while only the left fusiform gyrus showed a significantly positive correlation. In total scores of (both physical and mental) fatigue, the right SMC and orbital part of the inferior frontal gyrus (OIFG) showed a negative correlation, while the left putamen and MTG showed a positive correlation. Therefore, the right SMC may play a critical role in fatigue progression because of the only common factor among physical, mental, and total fatigue. The left putamen may play a compensatory role with a positive correlation to physical and total fatigue. Additionally, identifying the other gray matter regions that positively or negatively correlated to CFQ of healthy adults may help deepen the understanding of early-stage fatigue progression, leading to the future establishment of preventive measures through volumetrics by using MRI.

## Highlights

• The right supplemental motor cortex may play a critical role in the progression of physical, mental, and total fatigue.

## Graphical abstract

The impact of fatigue on the gray matter volume of healthy middle-aged adults shows color-coded with blue for negative correlation and red for positive correlation with Chalder's fatigue questionnaire (CFQ) scores on 3D brain images of axial, sagittal, and coronal sections.

**Figure:**
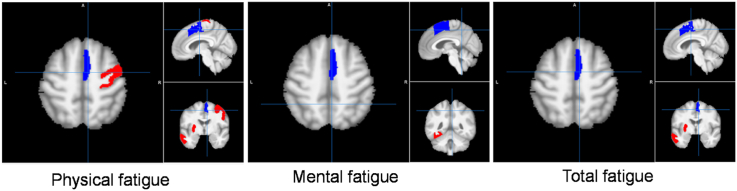

## 1 Introduction

Although a lot of people are suffering from chronic fatigue sometimes longer than 6 months, the integrated research on fatigue has not yet been organized before we organized the fatigue study group in Japan under the governmental organization of the Ministry of Education, Culture, Science, and Technologies (MEXT) (Watanabe et al., 2008). Fatigue is a sense that all people have ever experienced and is therefore intimate for us, but its molecular and neural mechanisms have not fully been elucidated yet, probably because of the complicatedness of the causes. However, we know the decrease in the efficiency of our tasks and studies by fatigue, and also the decrease in motivation for those. It is of great value in our society to extensively analyze the causes of fatigue and motivational loss, and to develop the quantification scales on fatigue and motivation for the invention of methods and therapies for better recovery and avoidance of severe chronic fatigue.

One of the typical diseases with a prolonged severe pathological state of chronic fatigue is known as Myalgic Encephalomyelitis/Chronic Fatigue Syndrome (ME/CFS). ME/CFS is a complex, chronic medical condition characterized by symptom clusters that include: pathological fatigue and malaise, especially post-exertional malaise, cognitive dysfunction, immune dysfunction, unrefreshing sleep, pain, autonomic dysfunction, neuroendocrine and immune symptoms (Zarrouf, 2008; Cope and David, 1996; Watanabe, 2021). ME/CFS is common, often severely disabling and costly. The Institute of Medicine (IOM) in US reviewed the ME/CFS literature and suggested a new name for ME/CFS and called it Systemic Exertion Intolerance Disease (SEID) (Jason et al., 2015). SEID's diagnostic criteria are less specific and do not exclude psychiatric disorders.

MRI morphometry studies with ME/CFS demonstrated the involvement in the bilateral prefrontal cortex (Watanabe et al., 2008), amygdala, insula (Finkelmeyer et al., 2018), and basal ganglia (De Lange et al., 2005). Structural neuroimaging studies with ME/CFS patients revealed frontal atrophy and volumetric changes of several brain regions including in the right hippocampal region and right dorsolateral prefrontal cortex (Barnden et al., 2011; Tang et al., 2015). On the other hand, functional imaging studies with normal healthy individuals have addressed the relationship between certain brain regions and the subjective feeling of acute fatigue (Suda et al., 2009; Cook et al., 2007; Tajima et al., 2010; Tanaka et al., 2006). In those studies, neural activity during attention-demanding tasks decreased in the ventrolateral prefrontal cortex (Suda et al., 2009) and posterior parietal cortex but increased in the cerebellar, temporal, cingulate, frontal (Cook et al., 2007), and medial orbitofrontal cortices (Tajima et al., 2010). Additionally, abnormal resting-state functional connectivity has been reported for left anterior mid-cingulate with the sensory-motor network (Gay et al., 2016). Furthermore, hypoperfusion was explored by many single photon emission computed tomography (SPECT) studies (Watanabe, 2021) and our previous positron emission tomography (PET) study (Kuratsune et al., 2002). Our PET study (Kuratsune et al., 2002) revealed a decrease in regional cerebral blood flow (rCBF) in the patients with ME/CFS in various brain regions including frontal, prefrontal, orbitofrontal, and insular cortices, middle occipital, middle temporal, anterior cingulate, parahippocampal, superior temporal, transverse temporal, and precentral gyri, putamen, globus pallidus, hippocampus, mesencephalon, and cerebellum. Nevertheless, the number of ME/CFS patients in the previous studies is relatively small, so that lacking the statistical power to explore precise brain regions with rather moderate changes or with rather smaller volumes.

Moreover, elucidation of the underlying neuronal mechanisms in fatigue progression can contribute to the early identification and prevention of chronicity. Since previous studies of fatigue mechanisms have been mainly performed using data from the patients with ME/CFS and not so enough number of the people in CF categories, the early or subclinical stage of fatigue progression remains still unclear in neuronal underpinnings. In the previous study, we demonstrated the gray matter volume (GMV) reduction in good correlation with a higher score of fatigue in 63 adults (age, 53.2 ± 8.3) (Kokubun et al., 2018), but we did feel the need of a larger-scale cohort study with healthy subjects.

In general, fatigue can be roughly categorized as a physical and mental one. Physical fatigue relates to muscle tiredness resulting from repeated actions (Van Duinen et al., 2007). Mental fatigue relates to cognitive tiredness because it is unable to complete mental or long-term cognitive tasks (Mizuno et al., 2011). Mental fatigue is also a psycho-biological state characterized by a feeling of tiredness and lack of energy (Marcora et al., 2009). Most studies investigating the relationship between physical and mental fatigue focus on how mental fatigue reduces physical performance and vice versa (Van Cutsem et al., 2017).

Therefore, in the present study, we performed voxel-based morphometry (VBM) analysis in 1873 healthy middle-aged adults with CFQ including physical and mental fatigue categories.

## 2 Materials and methods

### 2.1 Participants' profile

A total of 2910 individuals aged 20–89 underwent MRI and answered the questionnaire about fatigue as part of the brain healthcare checkups in Kochi Kenshin Clinic; affiliated with Kochi University of Technology. Generally, the main purpose of a brain healthcare checkup is to do a medical diagnosis. The diagnosis is done mainly using T1-and T2-weighted images and magnetic resonance angiography. Therefore, the present study was focused on brain structural data obtained by VBM analysis with T1-weighted imaging. Written informed consent was obtained from each subject of the projects in which they participated. The procedures for all studies were conducted in accordance with the Declaration of Helsinki and approved by the Ethics Committee of Kochi University of Technology.

**Fig. 1:**
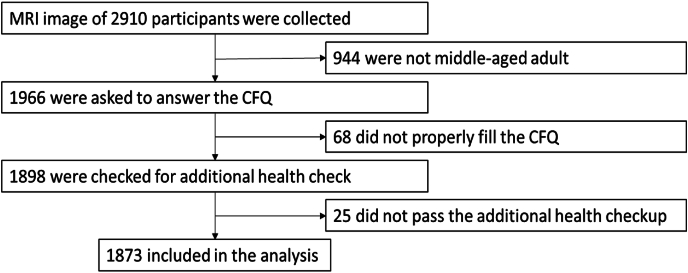
Participant's selection process.

The participants were enrolled under various conditions. Although they were instructed to stay still while in a supine position during the MRI scan, all images were not clear due to movement noises. Space-occupying lesions, such as brain tumors and arachnoid cysts, which disturb the volumetric measurement, were identified. Fig. 1 shows the participant's selection process for this study. Among the participants, only individuals right-handed and aged 45–65 years were selected; thus, 944 individuals were excluded. Handedness was evaluated using the Edinburgh Handedness Inventory (Chalder et al., 1993). All remaining participants were requested to answer the translated version of CFQ following the work of Tanaka (Tanaka et al., 2009). Sixty-eight individuals did not complete the CFQ. In relation to mental diseases, such as depression, cerebrovascular diseases (cerebral infarction), or multiple sclerosis, which may affect the CFQ or volumetric measurements, twenty-five individuals were excluded due to additional medical health checkups. Finally, 1873 participants (1040 males, 833 females; the age of 54.1 ± 5.4 years) were registered.

### 2.2 Assessment of fatigue

The degree of fatigue was evaluated with the Japanese-translated version of CFQ (Tanaka et al., 2009; Tanaka et al., 2008), a self-administered questionnaire used for measuring the extent and severity of fatigue for the clinical and non-clinical population. This questionnaire was initially developed to measure the degree of CF symptoms in clinical populations (Deale et al., 1997), and its scale was then revised, and it is now more widely used to measure tiredness in the non-clinical, general population (Chilcot et al., 2016; Cella and Chalder, 2010). The CFQ consists of 11 questions to calculate total fatigue score. The total fatigue score is divided into scores of 7-item physical fatigue and 4-item mental fatigue. The questions are non-threatening and are about sensation and functionality, rather than any beliefs or opinions about health status, which include the following: “Do you have problems starting things?” and “Do you have difficulty concentrating?” Each of the 11 items is answered on a 4-point scale (0, 1, 2, 3) ranging from asymptomatic to maximum symptomologies, such as “Better than usual,” “No worse than usual,” “Worse than usual,” and “Much worse than usual.” The total fatigue score for the 11-item scale ranges from 0 to 33, with higher scores indicating a greater degree of fatigue. For all items, the least symptomatic answers are on the left of the response set, providing an easy-to-understand checklist for respondents.

The reliability coefficients for CFQ were high in studies of patients with CFS (Morriss et al., 1998) and in occupational and general population research. CFQ has widely been used in studies about tiredness among working populations, and it is consistently and exceptionally good compared with other longer and multidimensional tools (De Vries et al., 2003). CFQ is widely used in occupational research, facilitating straightforward comparisons between studies and populations. This is considered as another advantage of CFQ.

### 2.3 Magnetic resonance imaging

T1-weighted MRI images were obtained using 1.5 T ECHELON Vega system (Hitachi, Tokyo, Japan) with the three-dimensional gradient-echo with inversion recovery (3D-GEIR) sequence. The following scanning parameters were used: repetition time, 9.2 ms; echo time, 4.0 ms; inversion time, 1000 ms; flip angle, 8°; the field of view, 240 mm; matrix size, 0.9375 × 0.9375 mm; slice thickness, 1.2 mm; and the number of excitations, 1. Each image was visually assessed for brain diseases and anomalies, head motion, and artifacts, which affect the volumetric measurement. The images were processed and analyzed using the VBM8 toolbox (http://dbm.neuro.uni-jena.de/vbm/) and other modules implemented in the Statistical Parametric Mapping (SPM) 8 (https://www.fil.ion.ucl.ac.uk/spm/) to estimate regional brain volumes.

In brief, the images were segmented into GM, WM, and cerebrospinal fluid space using the maximum a posteriori approach (Whitwell, 2009; Kurth et al., 2015). The segmented GM and WM images were then used to estimate the morphological correspondence between the template image and the participant's brain using the high-dimensional non-linear warping algorithm (Ashburner, 2007). The estimated non-linear warp was inversely applied to an atlas defined in the template space to anatomically parcellate the target brain. The Neuromorphometrics atlas incorporated in SPM12 was used for the parcellation, with a modification for WM lesions, which appeared as incorrect GM segments around the lateral ventricles. Each anatomical region volume was calculated as the sum of the correspondent tissue densities in the voxels belonging to each region.

### 2.4 Statistical analysis

The correlations among fatigue categories and brain volumes (overall volumes of gray matter and cerebrospinal fluid and total intracranial volume, including regional volumes for the 110 regions of the brain), as estimated using the SPM8 toolbox, were investigated using bivariate correlations in the Statistical Package for the Social Sciences software version 22 (IBM Corp., Armonk, NY, USA). Furthermore, an exploratory analysis of the related segment volume or rGMV to fatigue was also performed using stepwise multiple linear regression with 95% confidence intervals for each of the regional brain volumes. The stepwise multiple linear regression analysis was performed separately for each regional brain site and independently for each type of fatigue score. The three types of fatigue scores are physical fatigue scores, mental fatigue scores, and total fatigue scores. The total fatigue score represents all the participants and their complete CFQ item scores (physical + mental fatigue score). The physical, mental, and total fatigue scores were used as the dependent variables, whereas rGMVs were used as the independent variables. The linear regressions are corrected for age and sex with confidence intervals (CI) of 95%. Furthermore, residual analysis with a case-wise diagnostic for outliers outside three standard deviations was performed for all of the linear regressions. Additionally, age and sex (where the male is coded as 1 and female as 0) are added as covariate variables because they are widely known to affect brain volume (Gur et al., 2002). The volume data used were the rGMVs that had already been divided by the brain's intracranial volume. This was chosen to minimize the variation in brain sizes among individuals.

## 3 Results

### 3.1 Participants' profile

**Fig. 2:**
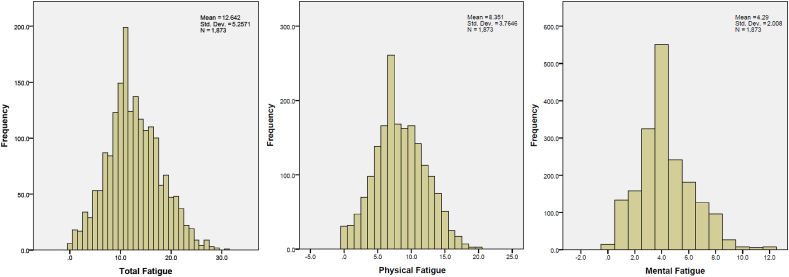
Distribution of the CFQ scores for total fatigue, physical fatigue, and mental fatigue.

Table 1 shows the mean and SD of the study participants for age, CFQ, and brain regions, including total brain volume, total gray matter volume (GMV), total white matter volume (WMV), and cerebrospinal fluid volume divided by intracranial volume (ICV). Fig. 2 shows the distribution of the CFQ scores for total fatigue, physical fatigue, and mental fatigue. The CFQ score was found to be highly reliable (11 items; Cronbach's *α* = 0.89).

**Table 1:** Age, Sex, CFQ score, and whole brain volumes corrected by intracranial volume in the participants.

	Mean	SD
Age	54.1	5.4
Sex (Female/Male)	833/1040	
CFQ		
Physical Fatigue Score	8.4	3.8
Mental Fatigue Score	4.3	2.1
Total Fatigue Score	12.6	5.3
Total BV/ICV	0.8232	0.0182
Total GMV/ICV	0.4261	0.0187
Total WMV/ICV	0.3971	0.0177
CSF/ICV	0.1767	0.0182

N = 1873. Abbreviations: CFQ, Chalder's Fatigue Questionnaire; BV, brain volume; GMV, gray matter volume; WMV, white matter volume; CSF, cerebrospinal fluid; ICV, intracranial volume; SD, standard deviation.

### 3.2 MRI data

#### 3.2.1 Analysis of WM volume

No significant correlations were found between CFQ scores and WM volume.

#### 3.2.2 Analysis of rGMV

Three-multiple regression analyses adjusted with sex and age were conducted to clarify the associations between 110 rGMVs and total fatigue, physical fatigue, or mental fatigue. The partial regression plots and a plot of studentized residuals against the predicted values showed evidence of linearity for all three regression analyses. The independence of residuals for total, physical, and mental fatigue were assessed by a Durbin-Watson statistic of 1.911, 1.936, and 1.887, respectively. A visual inspection of the plot of studentized residuals versus unstandardized predicted-values showed evidence of homoscedasticity for the three regressions. There was no evidence of multicollinearity, as assessed by tolerance values greater than 0.1. There were no studentized deleted-residuals greater than ±3 standard deviations, no leverage values greater than 0.2, and values for Cook's distance were above 1 for the three types of fatigue. The assumption of normality was met for the three regressions, as assessed by the Q-Q Plots. The multiple regression model predicted statistical significance for physical fatigue [F(8, 1864) = 15.526, p < .0005], mental fatigue [F(5, 1867) = 6.248, p < .0005], and total fatigue [F(6, 1866) = 14.752, p < .0005]. All variables presented in Table 2 added statistical significance to the prediction including age and sex parameters (p < .01).

**Table 2:** Multiple linear regression analysis results for physical fatigue, mental fatigue, and total fatigue score.

	Gray matter regions	Coefficient β	Std. Error	t value	*p*
CFQ score for Physical Fatigue	(1) Left putamen	0.701	0.251	2.796	**
	(2) Left middle temporal gyrus	0.239	0.114	2.089	*
	(3) Left parietal operculum	0.900	0.447	2.016	*
	(4) Right precentral gyrus	0.384	0.154	2.489	*
	(5) Right planum temporale	−2.015	0.581	−3.466	**
	(6) Right supplementary motor cortex	−1.069	0.300	−3.563	**
CFQ score for Mental Fatigue	(7) Left fusiform gyrus	0.324	0.129	2.502	*
	(8) Left lateral orbital gyrus	−1.096	0.383	−2.860	**
	(6) Right supplementary motor cortex	−0.415	0.151	−2.745	**
CFQ score for Total Fatigue	(1) Left putamen	0.968	0.350	2.764	**
	(2) Left middle temporal gyrus	0.324	0.158	2.046	*
	(9) Right orbital part of the inferior frontal gyrus	−2.415	1.023	−1.251	*
	(6) Right supplementary motor cortex	−1.206	0.393	−3.070	**

Three separate multiple linear regressions, one for each fatigue type were done.

The three regressions used age, sex, and 110 regions of the brain as the independent variables (IV).

Final IVs were selected using the stepwise method.

Both age and sex have a statistically significant negative correlation with all three fatigue scores.

Red figures show negative correlations.

N = 1873; *p < .05; **p < .01.

**Fig. 3:**
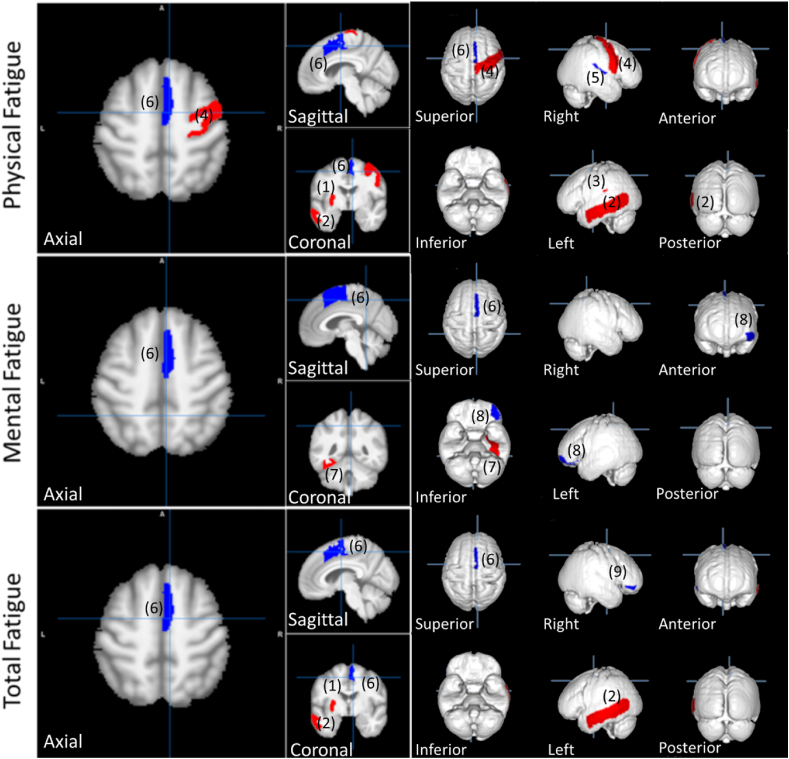
Impact of fatigue on the gray matter volume of the participants based on Table 2. Color-coded with blue for negative change and red for positive change. (1) left putamen, (2) left middle temporal gyrus, (3) left parietal operculum, (4) right precentral gyrus, (5) right planum temporale, (6) right supplementary motor cortex, (7) left fusiform gyrus, (8) left lateral orbital gyrus, (9) right orbital part of the inferior frontal gyrus. (For interpretation of the references to color in this figure legend, the reader is referred to the Web version of this article.)

The result of the statistical analyses shows that 6 Gy matter regions were significantly correlated with physical fatigue score, three regions for mental fatigue, and four regions for total fatigue (Table 2). The brain regions that are significantly correlated with the fatigue scores are shown in Fig. 3. The figure is color-coded with blue and red to represent the negative correlation and positive correlation, respectively. Positive correlation denotes that the affected rGMV is bigger with a high CFQ score, while negative correlation denotes a smaller volume for the affected rGMV with a high CFQ score. Scatter plots of each significant rGMVs are illustrated in the additional materials. The details of the result are discussed in the discussion section.

## 4 Discussion

Previously, we showed the gray matter volume reduction in parallel with the subjective scores of fatigue in the healthy adult population (Kokubun et al., 2018). However, the number of volunteers (63) was not so large. As far as we know, there is a study using a larger number of normal healthy individuals. The Tohoku University research team investigated 883 university students with the age of 20.7 ± 1.81 (Nakagawa et al., 2016). They demonstrated no significant correlation between rGMV with fatigue severity. However, the mean diffusivity (MD) values describing microstructural properties with diffusion tensor imaging in the right putamen, pallidus, and caudate significantly correlated with the degree of fatigue and motivation, whereas physical activity significantly correlated with the MD values of the right putamen. The authors proposed a plausible mechanism underlying the development of fatigue: the tendency to develop fatigue might be correlated with the physical process as well as the mental one via the dopaminergic system functioning in the basal ganglia. However, it is not yet known what happens afterward (path to the chronic fatigue state), and is also speculative that the middle-aged subjects tend to overwork rather than the younger people around the age of 20.

On the other hand, in the present study, we identified GM regions volumetrically correlating to CFQ scores, using a large sample of 1873 healthy adults with a mean age of 54.1 ± 5.4 (Table 2). One reason is that middle-aged adults may be more frequently exposed to fatigue and more vulnerable to recovery from fatigue than young adults around 20 years old at Tohoku University study (Nakagawa et al., 2016). Another is that the same MRI equipment in the present Kochi study had been used in the same scanning conditions, resulting in the minimum of measure biases. Under the present circumstance in Japanese clinics and hospitals, it has an advantage in that VBM using 1.5T MRI as a part of the brain health check-ups is more prevalent and feasible for large amounts of data than functional analyses using 3T MRI.

Based on the statistical analysis presented in Table 2, we found that the right SMC is negatively involved not only with physical fatigue but also with mental and total fatigue. This suggests that the people with higher fatigue scores have a smaller volume of the right SMC despite different causes of fatigue. The finding is consistent with previously reported functional imaging studies about patients with ME/CFS (Filippi et al., 2002). To find out which physical or mental fatigue is more correlated with the right SMC, an additional multiple linear regression was conducted. The right SMC volume was used as the independent variable and physical and mental fatigue scores as the dependent variable. The result indicates that the mental fatigue score is significantly correlated with the right SMC (p < .05; data not shown), while the physical score is not significantly correlated with one. The SMC has been reported in various functional MRI imaging studies to be involved in the retrieval of motor memory, limb planning movements, and temporal organization of movements (Van Cutsem et al., 2017; Halsband et al., 1993; Makoshi et al., 2011). These processes inherent in movements are related to cognitive activity in addition to physical activity. This may be one of the reasons why the right SMC significantly correlates with the mental fatigue score although the result does not come from functional imaging analyses, but from volumetric measurements.

The planum temporale (PT) is involved in early-phase auditory processing, specifically in the discrimination and selection of stimuli from the left and right ears (Binder et al., 1996). When left and right PT located with anatomical brain scans were stimulated with repetitive transcranial magnetic stimulation (rTMS), rTMS over the right but not left PT significantly reduced the right ear advantage. The result revealed that the right planum temporale is involved in auditory selection and attention (Hirnstein et al., 2013). In the present study, right PT is negatively involved with physical fatigue. Deterioration in early-phase auditory processing may be possibly explained as a precedent factor of fatigue development.

The left putamen was significantly positive in physical and total fatigue, but not in mental fatigue. The putamen is involved in the regulation of movement and learning, including speech articulation, reward,

Cognitive functioning, and addiction (Haruno and Kawato, 2006; Ghandili and Munakomi, 2021). Tohoku University study also showed functional abnormalities

In the putamen and caudate operating as a dopaminergic system (Nakagawa et al., 2016). Both the Tohoku University study and the present study targeted healthy individuals. Putamen may positively interact with physical fatigue, that is, become larger in volume as one of the defenses against fatigue development except for mental processes since biological reactions often induce a compensatory process. Usually, volume reduction is due both to neuronal cell loss and to less arborization of dendrites. The latter is actually a reversible phenomenon by rehabilitation and/or some anti-inflammatory drugs. In the case of ME/CFS, we found the rGMV reduction in bilateral prefrontal cortices and published the results in BMC Neurology in 2004 (Okada et al., 2004). Then, the results were reproduced by the other group and then they found the recovery of this volume loss in the responder's group by 6-month cognitive behavioral therapy (de Lange et al., 2005).

According to Watanabe (Watanabe, 2021), the fluctuation of some of the rGMVs with subjective sensation of fatigue suggests activation of defense mechanisms in the pathophysiology of ME/CFS in order to avoid further exhaustion. This statement is supported by small-scale studies of healthy individuals in healthy state (Tanaka et al., 2015) as well as in ME/CFS pathological states. The same hypothesis may be true in the positive correlation to the left MTG, left parietal operculum, and right precentral gyrus to severity scores of fatigue. Functional and metabolic imaging studies with a large scale of healthy individuals will be needed to validate this hypothesis.

The left MTG was also significantly positive only in physical and total fatigue. The left MTG is known to be involved in visual recognition and understanding of words while reading. Dysfunction in MTG may lead to alexia and agraphia (acquired impairment affecting reading and writing ability) (Suh et al., 2010; Sakurai et al., 2010). The positive correlation of the MTG volume to fatigue has been reported for the first time but needs to be further investigated whether the brain mechanism of fatigue development is involved in that of alexia and agraphia.

The right precentral gyrus and left parietal operculum were also significantly positively proportional to physical fatigue scores. The precentral gyrus belongs to the primary motor cortex and it is suggested that the right precentral gyrus merges oculomotor and somatomotor space coding in the human brain (Iacoboni et al., 1997). The parietal operculum that forms the ceiling of the lateral sulcus functions as the secondary somatosensory cortex. In summary, all the regions described above are possibly explained as correspondence in the brain to feel dully through fatigue development.

In mental fatigue, left OIFG was significantly negative. Left OIFG plays an important role in emotional and motivational decision-making, which possibly explains the involvement in fatigue development. In contrast, a significant positive reaction was observed in the fusiform gyrus, which entails higher processing of visual information, including the identification and differentiation of objects (Furl et al., 2011). The positive reaction of the fusiform gyrus may play a compensatory role in fatigue development similar to the left putamen, MTG, and parietal operculum, and to the right precentral gyrus.

Thus, the identification of several brain regions negatively or positively correlating to fatigue degree may contribute to the early diagnosis of chronic fatigue even in some cases of ME/CFS or evaluation of the preclinical state in healthy middle-aged individuals. Physical malfunction via right SMC and mental compensatory via left putamen may be simply explained as the early mechanism of fatigue development. Volumetric data using MRI may be an alternative to questionnaire scores where the arbitrary effect cannot be denied although MRI examination is highly expensive.

Our study has the following limitations; Although the CFQ score was used for examining fatigue degrees because of a simple and widely used score, the CFQ score has its own limitation due to subjective awareness of fatigue. The present study showed an important but low correlation between fatigue and rGMV. It may increase when other fatigue measurements are used together in addition to the CFQ score. This study was also conducted at a single center in Kochi prefecture in Japan, which means that we have to take into consideration of selection bias. We are much interested in the early stage of fatigue progression and used healthy subjects with CFQ scores, which must be a big limitation for ME/CFS studies. Based on the evidence with CFQ scores, we will proceed to investigate the mechanism of chronicity and pathogenesis for ME/CFS using other metrics such as biological oxidation (Norheim et al., 2011), autonomic nerve function (Freeman and Komaroff, 1997), and neuroinflammation (Lee and Giuliani, 2019) in the near future. In addition, the present work is a cross-sectional study with 1.5T MRI from a very specific healthcare center in Japan. Almost all healthcare centers use 1.5T MRI in Japan. The use of 1.5T MRI is the evitable limitation for the present study. Based on the study results, we will proceed to conduct the 3T MRI research. Furthermore, this study focuses on finding a correlation between rGMV and fatigue, of course, the correlation does not prove any causes. In order to improve and support the present results, longitudinal data are needed for elucidating causal relationships in our future study. Since the present study focused on the middle-aged group, it is worth noting that the fatigue score for the female group might be affected by menopause. Now we are planning to investigate such a point including the sex difference in cohort design studies. Such analyses seem quite possible to happen since we can steadily collect MRI data over time through brain healthcare check-ups that are largely prevalent in Japan as preventive medicine. Then, we may be able to validate the neuronal underpinning in fatigue development and identify individuals who are at risk of chronicity using brain volumetric data which requires less time and effort than brain functional data. The research strategy may not only provide effective means of reducing socio-economic losses due to fatigue but also help prevent death from overwork, Karoshi (Kanai, 2009), which is one of the serious and urgent healthcare problems for middle-aged workers in Japan (Kondo and Oh, 2010).

## Contributors

KP designed the study and collected the participants’ data. FY design the rGMV calculation using VBM. KP and HP had full access to raw data. HP carried out the final statistical analyses and prepared all the figures and tables. KP, KM, and YW accessed and verified the underlying data. HP wrote the first draft of the manuscript. HP, KP, KM, and YW reviewed and revised the manuscript to completion. All authors had final responsibility for the decision to submit for publication.

## Data sharing

After publication, data will be available to any researcher who provides a methodologically sound study proposal that is approved by the central study team. Individual participants will not be identifiable in any released data and all appropriate information governance.

## Declaration of competing interest

The authors declare that they have no known competing financial interests or personal relationships that could have appeared to influence the work reported in this paper.
