# Supplementary material for: Regional gray matter volume correlates to physical and mental fatigue in healthy middle-aged adults
Source: Neuroimage Rep. 2022 Sep 2;2(4):100128. doi: 10.1016/j.ynirp.2022.100128 (PMC12172783; doi:10.1016/j.ynirp.2022.100128)
Supplement: Multimedia component 1 [file mmc1.docx]

Appendix, additional materials 1


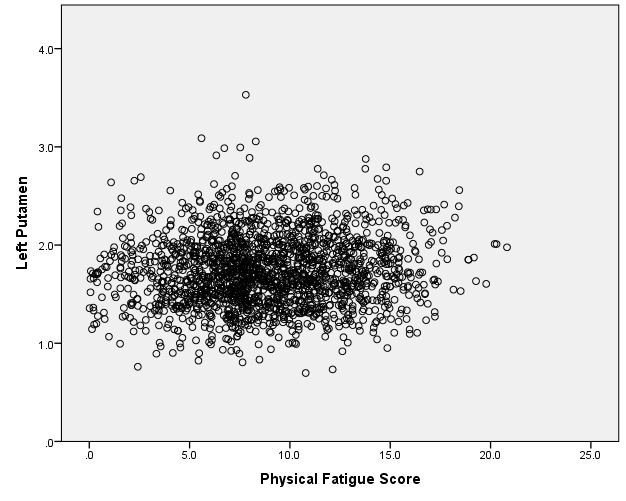

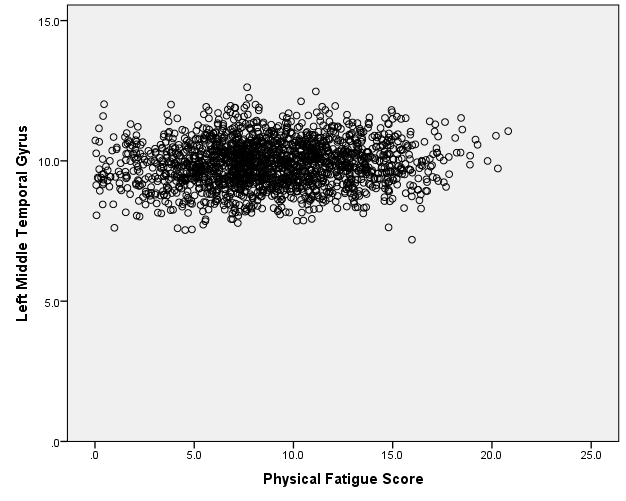

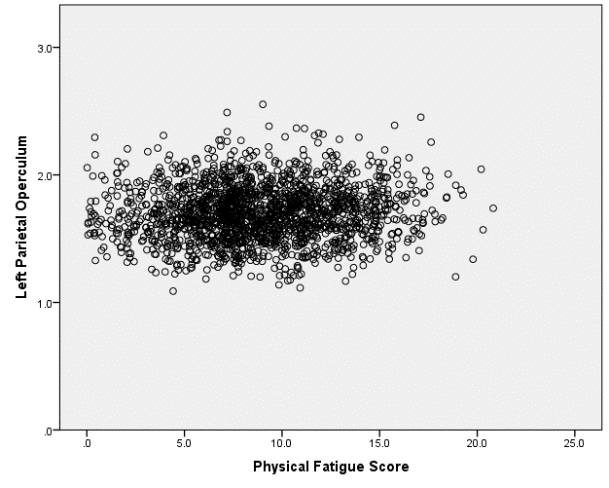

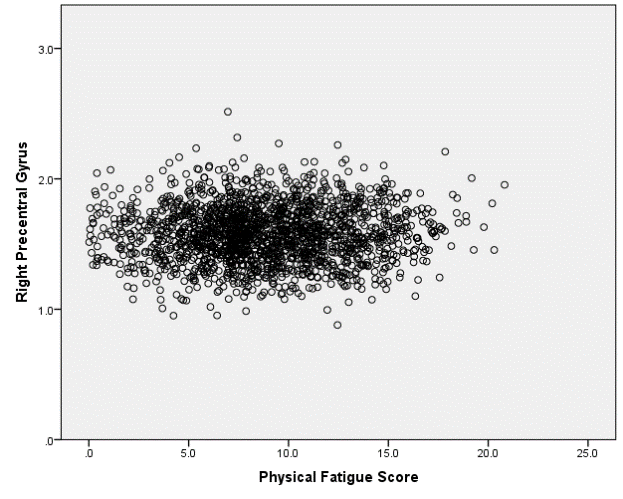

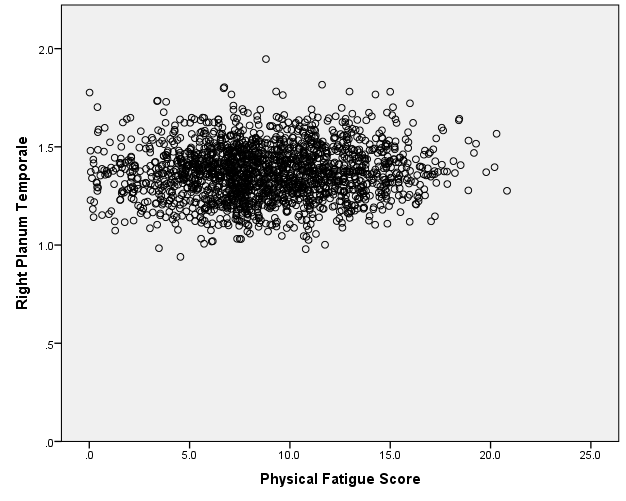


Scatter plot of rGMV vs Physical Fatigue Score, showing the distribution of the regional brain volume for physical fatigue score. (The physical fatigue score was added with a random number with a mean of 0 and a standard deviation of 1).

Appendix, additional materials 2


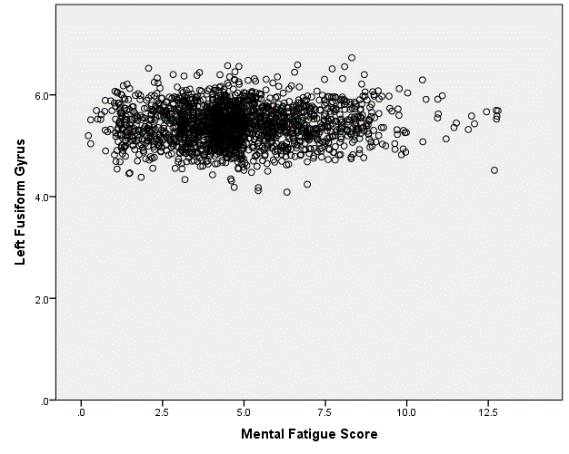

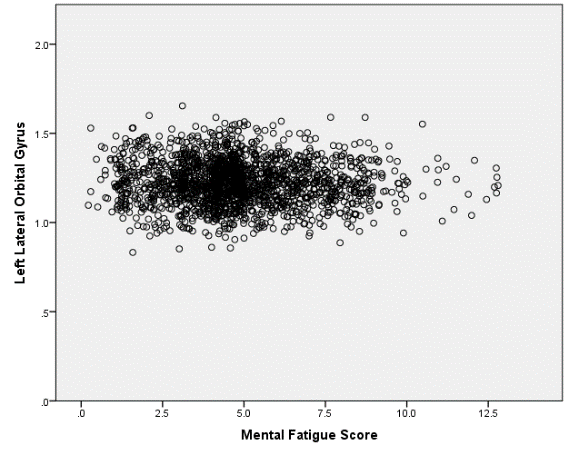

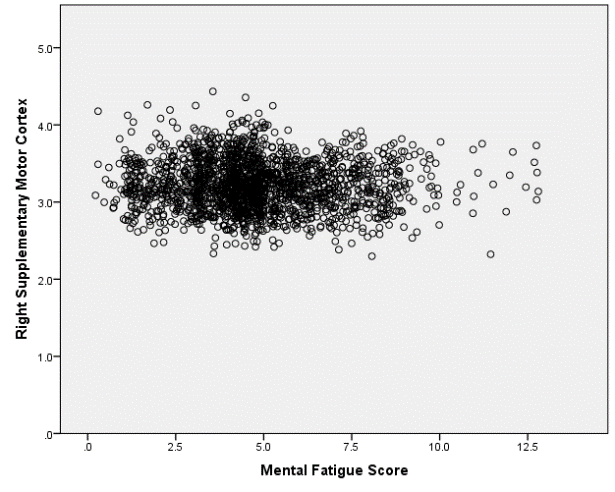


Scatter plot of rGMV vs Mental Fatigue Score, showing the distribution of the regional brain volume for mental fatigue score. (The mental fatigue score was added with a random number with a mean of 0 and a standard deviation of 1).

Appendix, additional materials 3


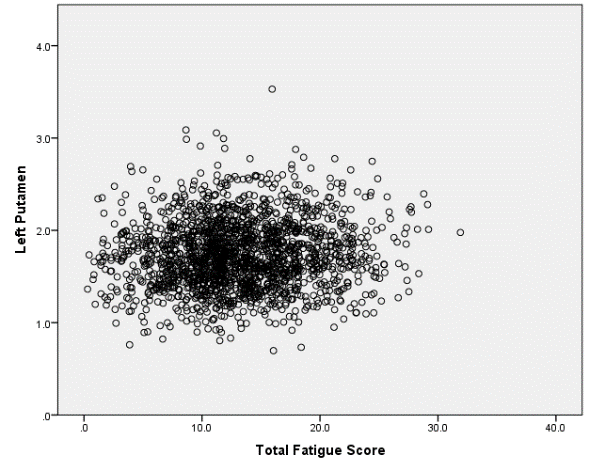

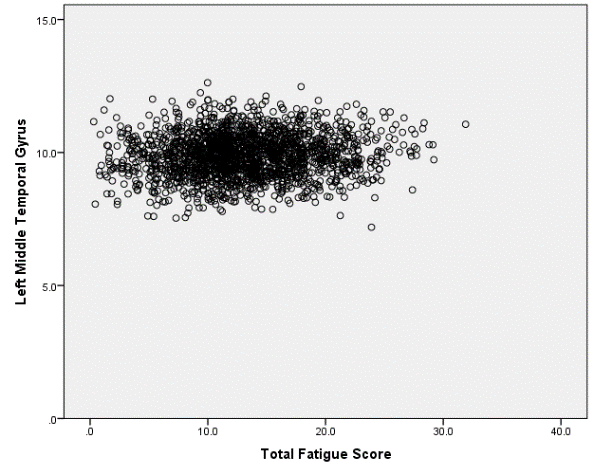

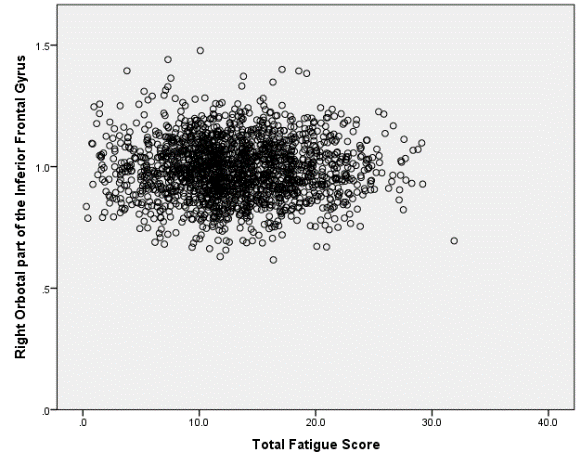

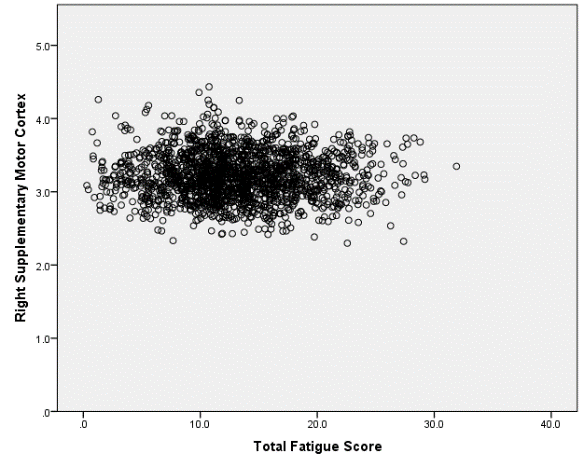


Scatter plot of rGMV vs Total Fatigue Score, showing the distribution of the regional brain volume for total fatigue score. (The total fatigue score was added with a random number with a mean of 0 and a standard deviation of 1).
